# Association of Environmental Cadmium Exposure with Periodontal Disease in U.S. Adults

**DOI:** 10.1289/ehp.0800312

**Published:** 2009-01-22

**Authors:** Manish Arora, Jennifer Weuve, Joel Schwartz, Robert O. Wright

**Affiliations:** 1 Population Oral Health, Faculty of Dentistry, Westmead Centre for Oral Health, University of Sydney, Sydney, New South Wales, Australia;; 2 Environmental and Occupational Medicine and Epidemiology, Department of Environmental Health, Harvard School of Public Health, Boston, Massachusetts, USA;; 3 Rush Institute for Healthy Aging, Rush University Medical Center, Chicago, Illinois, USA;; 4 Channing Laboratory, Department of Medicine, Brigham and Women’s Hospital, Harvard Medical School, Boston, Massachusetts, USA

**Keywords:** environmental tobacco smoke, NHANES III, periodontal disease, smoking, urine cadmium

## Abstract

**Background:**

Periodontal disease is a complex, multifactorial, chronic inflammatory disease that involves degradation of periodontal structures, including alveolar bone. Cadmium adversely affects bone remodeling, and it is therefore possible that environmental Cd exposure may be a risk factor for periodontal-disease–related bone loss.

**Objective:**

We examined the relationship between environmental Cd exposure and periodontal disease in U.S. adults.

**Methods:**

We analyzed cross-sectional data from the third National Health and Nutrition Examination Survey (NHANES III). We defined periodontal disease as clinical attachment loss of at least 4 mm in > 10% of sites examined. We used multivariable-adjusted logistic regression analyses to estimate the association between creatinine-corrected urinary Cd levels and periodontal disease.

**Results:**

Of the 11,412 participants included in this study, 15.4% had periodontal disease. The age-adjusted geometric mean urine Cd concentration (micrograms per gram creatinine) was significantly higher among participants with periodontal disease [0.50; 95% confidence interval (CI), 0.45–0.56] than among those without periodontal disease (0.30; 95% CI, 0.28–0.31). Multivariable-adjusted analyses, which included extensive adjustments for tobacco exposure, showed that a 3-fold increase in creatinine-corrected urinary Cd concentrations [corresponding to an increment from the 25th (0.18 μg/g) to the 75th (0.63 μg/g) percentile] was associated with 54% greater odds of prevalent periodontal disease (odds ratio = 1.54; 95% CI, 1.26–1.87). We observed similar results among the subset of participants who had limited exposure to tobacco, but only after removing six influential observations.

**Conclusion:**

Environmental Cd exposure was associated with higher odds of periodontal disease.

Periodontal disease is a common, multi- factorial, chronic inflammatory disease that involves degradation of tissues that support teeth, including alveolar bone (jaw bone) (Pihlstrom et al. 2005). Colonization by predominantly gram-negative bacteria arising from dental plaque stimulates an inflammatory response that in some individuals, results in the breakdown of the connective tissue surrounding teeth (Preshaw et al. 2004). Periodontal disease can lead to tooth loss in adults and has been linked to a number of systemic disorders such as cardiovascular disease, stroke, and preterm birth (Pihlstrom et al. 2005). Although environmental factors have been implicated as possible risk factors (Barbour et al. 1997; Saraiva et al. 2007), the role of environmental toxins in the etiology of this disease has received limited attention.

Cadmium is a ubiquitous toxicant in our environment, and an estimated 2.3% of the U.S. population has elevated levels of urine cadmium (> 2 μg/g creatinine), a marker of chronic exposure and body burden (Paschal et al. 2000). Human exposure to Cd is possible from a number of sources, with smoking being a major contributor (Paschal et al. 2000). Other sources of Cd include emissions from industrial activities, including mining, smelting, and manufacturing of batteries, pigments, stabilizers, and alloys [Agency for Toxic Substances and Disease Registry (ATSDR) 2008]. Cadmium is also present in trace amounts in certain foods such as leafy vegetables, potatoes, grains and seeds, liver and kidney, and crustaceans and mollusks (Satarug et al. 2003). Once in the body, Cd accumulates in the kidney and liver and is excreted slowly over several years (ATSDR 2008).

The pathologic effects of Cd on bone, including the ability to promote inflammation, are pertinent to periodontal disease, where disruption of the host inflammatory response is considered a primary factor in disease progression and subsequent alveolar bone loss. The adverse effects of Cd exposure on bone are well established (Järup 2003; Kazantzis 2004). Several epidemiologic studies have documented an association of chronic low-level Cd exposure with decreases in bone mineral density (BMD) and osteoporosis (Åkesson et al. 2006; Alfvén et al. 2000; Gallagher et al. 2008; Honda et al. 2003; Schutte et al. 2008; Staessen et al. 1999). Data from experimental models suggest that Cd disrupts the bone remodeling process, decreases skeletal mineralization, and enhances bone loss (Brzóska and Moniuszko-Jakoniuk 2004). Cadmium exposure has also been shown to stimulate, in a number of cell types and tissues, the production of prostanoids (e.g., prostaglandin E2), cytokines [e.g., interleukins, tumor necrosis factor-α (TNF-α)], and matrix metalloproteinases (MMP) (Hemdan et al. 2006; Kirschvink et al. 2005; Marth et al. 2000, 2001; Romare and Lundholm 1999; Suzuki et al. 1989).

Based on this evidence, our hypothesis was that, because of its ability to promote inflammation and affect the bone remodeling process, environmental Cd exposure may be associated with periodontal disease. We used data from the third National Health and Nutrition Examination Survey (NHANES III), a nationally representative survey conducted from 1988 through 1994 in the United States. We placed particular emphasis on accounting for exposures to environmental tobacco smoke (ETS), which may potentially confound the observed association between Cd and periodontal disease.

## Materials and Methods

### Study participants

NHANES III identified 39,695 persons for sampling, of whom 33,994 were interviewed and 30,818 attended the mobile examination center [National Center for Health Statistics (NCHS) 1996]. Dentate individuals who were ≥ 13 years of age and who lacked medical contraindications (*n* = 28,059) were eligible for periodontal assessments (Westat, Inc. 1992). For the present study, we were interested in adult periodontal disease and included only participants ≥ 18 years of age (*n* = 17,752). Among these, we excluded participants who had no periodontal assessment or a partial assessment (*n* = 3,347) or invalid laboratory measurements of urine Cd (*n* = 912) or of serum cotinine (a biomarker of exposure to ETS; *n* = 1,555). We further excluded pregnant (*n* = 264) and breast-feeding women (*n* = 100) to avoid the effects of increased bone remodeling and also transient changes to periodontal tissues during these periods (Kalkwarf and Specker 2002; Laine 2002; Sowers 1996). Overall, our study population included 11,412 participants.

### Urine Cd and creatinine measurements

Details of the urine collection and Cd analysis have been reported previously (Paschal et al. 2000). Briefly, during the physical examination, a 10-mL spot sample of urine was collected from survey participants. Cadmium was quantified by Zeeman effect graphite furnace atomic absorption spectrophotometry with a detection limit of approximately 0.01 ng/mL (NCHS 2006). Each sample was analyzed in duplicate, and mean measurements were reported. Specimens with Cd concentrations > 6 ng/mL were reanalyzed for confirmation (Paschal et al. 2000). We used creatinine-corrected urine Cd concentrations (micrograms Cd per gram of creatinine) in our analyses.

### Periodontal examination

**Figure 1:**
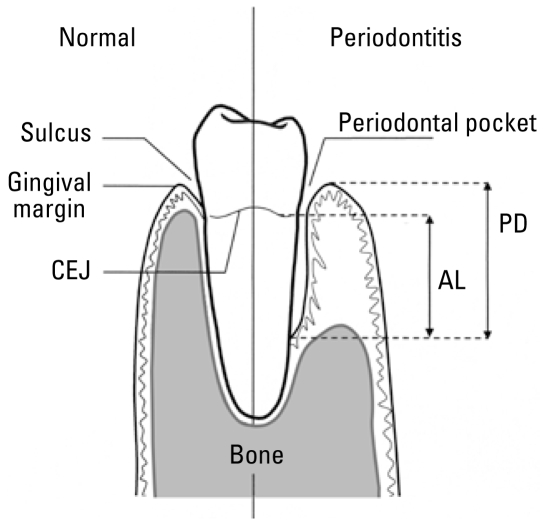
Diagram comparing healthy periodontal tissues with those affected by periodontal disease. In NHANES III, clinical attachment loss (AL) was the distance from the CEJ to the base of the sulcus measured in millimeters using an NIDR periodontal probe. We calculated this distance by subtracting from the pocket depth (PD) the distance from the gingival margin to the CEJ.

Details of the periodontal examinations have been previously described (Westat, Inc. 1992). Briefly, six trained and calibrated dentists examined a maximum of seven teeth of the upper jaw in a randomly selected half-mouth (quadrant). Similarly, a maximum of seven lower teeth were also examined in a randomly selected lower quadrant, giving a maximum possible total of 14 teeth examined per participant. Clinical measurements of periodontal disease were done at two sites (mesiobuccal and buccal) for each tooth. We focused our analysis on clinical attachment loss, which is a measure of the amount of periodontal tissue lost because of the disease process. In NHANES III, clinical attachment loss was the distance from the cementoenamel junction (CEJ) to the base of the sulcus measured (in millimeters) using a National Institute of Dental Research (NIDR) periodontal probe (Figure 1). We calculated this distance by subtracting from the pocket depth, the distance from the gingival margin to the CEJ. We used a case definition of periodontitis as attachment loss of ≥ 4 mm in > 10% of sites examined.

### Assessment of tobacco exposure

Because cigarette smoke is a major source of Cd in U.S. adults (Paschal et al. 2000) and is also associated with an increased risk of periodontal disease (Tomar and Asma 2000), we considered it an important potential confounder of any observed association between Cd exposure and periodontal disease. We incorporated into our study two assessments of cigarette smoking. First, we used serum cotinine, a biomarker of both active and passive exposure to cigarette smoke. Serum cotinine was measured using isotope-dilution liquid chromatography with tandem mass spectrometry (Bernert et al. 1997), with a limit of detection of 0.05 ng/mL (NCHS 2006). Second, we used pack-years of cigarette smoking, calculated by multiplying the reported average number of cigarettes smoked daily by the reported number of years smoked and dividing this product by 20 (cigarettes per pack). In addition to cigarette smoking, we used questionnaire-recorded responses to categorize participants as never, past, or current users of chewing tobacco, pipes, and cigars. To estimate ETS exposure, we used, in addition to serum cotinine, the response to a questionnaire item: “At work, how many hours per day are you close enough to people who smoke so that you can smell the smoke?” To account for potential exposure to ETS at home, we included the number of smokers at the participant’s home (0 vs. ≥ 1) in our analyses.

### Diabetes, BMD, and urinary albumin measurements

We included in our analyses certain systemic conditions that may be potential confounders because of their reported associations with both Cd exposure and periodontal disease. We classified a participant as having type 2 diabetes if he or she reported a physician diagnosis of diabetes (other than during pregnancy), reported taking prescription medications (either insulin or oral agents) for diabetes, had a nonfasting plasma glucose ≥ 11.1 mmol/L (200 mg/dL), had a fasting plasma glucose ≥ 7.0 mmol/L (126 mg/dL), or had a glycosylated hemoglobin (HbA_1c_) measurement > 6.1% (Perlstein et al. 2008; Rohlfing et al. 2000).

Because studies have previously reported associations between osteoporosis and periodontal disease (Wactawski-Wende 2001), we also included in our analyses total hip BMD measurements to adjust for osteopenia and osteoporosis. NHANES III measured BMD using a dual-energy X-ray absorptiometry instrument (QDR 1000; Hologic, Waltham, MA, USA), and verified data quality in a quality control program (Wahner et al. 1994). Urinary albumin concentrations were measured using a solid-phase fluorescent immunoassay (Gunter et al. 1996). Urinary albumin concentrations were dichotomized at 30 μg/mL because concentrations above this level are considered indicative of renal damage (Paschal et al. 2000).

### Statistical analysis

We examined summary statistics and scatter plots for key variables to identify outliers. We also studied age-adjusted geometric mean urine Cd concentrations [age standardized to the 2000 U.S. census population using age categories of 18–39, 40–59, and ≥ 60 years (Klein and Schoenborn 2001)] and periodontitis within key subject characteristics. Subsequently, we used multivariable-adjusted logistic regression to estimate the prevalence odds ratio (OR) of having periodontal disease for increments in log_e_-transformed, creatinine-corrected urinary Cd concentration. We report our results as ORs for a 3-fold increase in urinary Cd [i.e., exp(β̂_log_e_[_*_Cd_*_]_ × ln[3])], which corresponds to an increment in urine Cd concentration from the 25th to the 75th percentile. The principal models incorporated variables previously associated with periodontal disease, including age (years), sex, education of head of household (< high school, some high school, or > high school), ethnicity (non-Hispanic white, non-Hispanic black, Mexican American, other), household income (≥ 100% of federal poverty line or < 100% of federal poverty line), serum cotinine (nanograms per milliliter), cigarette smoking (0, > 0 to 9.9, ≥ 10 pack-years), chewing tobacco (never, past, current user), pipe smoking (never, past, current user), cigar smoking (never, past, current user), number of smokers at home (0 vs. ≥ 1), exposure to ETS at work (yes vs. no), number of teeth missing, BMD (grams per square centimeters), urine albumin (≤ 30 μg/mL vs. > 30 μg/mL), blood lead level (micrograms per deciliter), and type 2 diabetes (yes vs. no). We included survey phase and examiner identification numbers (coded 1 to 6) in the model to adjust for differences between survey phases and variations in the measurements of periodontitis (Slade and Beck 1999).

We were concerned that the observed association between Cd and periodontal disease may be confounded by active or passive exposure to tobacco. We therefore undertook parallel analyses restricted to participants with limited tobacco exposure (never-users of cigarettes, pipes, cigars, and chewing tobacco; no reported smoker at home; no reported exposure to smoke at work; serum cotinine concentrations ≤ 10 ng/mL). Furthermore, we were also concerned that the markers of systemic conditions (diabetes, BMD, urinary albumin) we included in our analyses may not be confounders but rather intermediates in the relation of Cd exposure to periodontal disease. We, therefore, undertook additional analyses excluding these variables from our logistic regression model to determine if their exclusion would alter the observed association between Cd and periodontal disease. We also excluded missing teeth variables from this analysis because tooth loss may be a consequence of periodontal disease rather than a confounder. Blood lead has previously been associated with periodontal disease, so we undertook analyses restricted to participants with blood lead concentrations < 3 μg/dL, which was the median blood lead concentration in our participants. For all data analyses, we used SAS version 9.1 (SAS Institute Inc., Cary, NC, USA) and accounted for the complex multistage sampling design of NHANES III. For our multivariable-adjusted logistic regression analyses, we used DFBETA (or Δβ) to identify observations that unduly influenced the parameter estimates for creatinine-corrected urinary Cd concentration [see Supplemental Material for details (http://www.ehponline.org/members/2009/0800312/suppl.pdf)]. We identified influential points separately in models for the full cohort and the low-tobacco group and provide results for analyses that excluded these observations as well as results when we retained these data.

## Results

The median creatinine-corrected urine Cd concentration of the participants in our study was 0.34 μg/g creatinine, with the 5th and 95th percentiles being 0.03 and 1.37 μg/g creatinine, respectively. The participants in our study were 40.9 years of age, on average (standard deviation = 17.2), and 52.3% were female. Approximately 15% (*n* = 1,758) of participants exhibited periodontal disease, as defined by the presence of attachment loss of at least 4 mm in > 10% of sites examined. Urinary Cd levels tended to be higher among participants who were female, were older, had lower levels of formal education, were of non-Hispanic black race/ethnicity, or had a household income below the federal poverty line (Table 1). Indicators of exposure to tobacco—including cigarette smoking, serum cotinine, and exposure to cigarette smoke at home and work—were positively associated with age-adjusted urinary Cd concentrations. We observed no remarkable associations between age-adjusted urinary Cd concentrations and pipe, cigar, or chewing tobacco use. Blood lead, urine albumin, having type 2 diabetes, and missing teeth were positively associated with urinary Cd concentrations, whereas BMD was inversely associated with urine Cd. Notably, participants with periodontal disease had significantly higher age-adjusted urinary Cd concentrations than those without periodontal disease (Table 1).

Similar to another report of periodontal disease in NHANES III (Borrell et al. 2005), cases of periodontal disease were significantly more prevalent among participants who were male, were older, had less formal education, or had a lower household income (data not shown). A significantly higher proportion of current smokers had periodontal disease compared with past- or never-smokers, and the prevalence of periodontal disease was also significantly higher with increasing serum cotinine concentrations (data not shown).

Using multivariable-adjusted logistic regression analyses, we observed that, for a 3-fold increment in creatinine-corrected urinary Cd concentrations [equivalent to the increment from the 25th (0.18 μg/g creatinine) to the 75th (0.63 μg/g creatinine) percentile in urinary Cd], the odds of having periodontal disease increased by 54% [OR = 1.54; 95% confidence interval (CI), 1.26–1.87; Table 2]. In this model, increasing age, male sex, having less than high school education, non-Hispanic black race/ethnicity, and household income below the federal poverty line were also significantly associated with increased odds of having periodontal disease. Participants who indicated up to 9.9 pack-years of smoking had increased odds for periodontal disease (OR = 1.67; 95% CI, 1.06–2.61) compared with never-smokers, an association similar to that for participants reporting 10 or more pack-years of smoking (OR = 1.44; 95% CI, 0.92–2.26). Diabetes and having missing teeth were also associated with increased odds of periodontitis. As expected, when all tobacco variables were excluded from our analyses, the association between urinary Cd and periodontal disease was notably stronger (OR = 1.87; 95% CI, 1.54–2.26).

In analyses restricted to participants with blood lead concentrations < 3 μg/dL, the association between urine Cd concentrations and periodontal disease remained significant (OR = 2.09; 95% CI, 1.39–3.13). In other analyses that excluded diabetes, BMD, renal dysfunction, and number of missing teeth, the association between Cd and periodontal disease remained nearly identical to the fully adjusted results (per tripling in creatinine-corrected urinary Cd: OR = 1.52; 95% CI, 1.25–1.85; Table 2). When the analysis was restricted to participants with low tobacco exposure, a 3-fold increase in urinary Cd concentrations corresponded to a 68% increase in the odds of having periodontal disease (OR = 1.68; 95% CI, 1.26–2.24).

In our multivariable-adjusted logistic regression analyses, we found that some observations unduly influenced the parameter estimate for creatinine-corrected urine Cd concentrations. In analyses of data from our whole study population, we identified five observations that were deemed to be influential data points. When these observations were retained in the model, the association between urine Cd and periodontal disease remained significant (OR = 1.32; 95% CI, 1.06–1.64). In our analyses of participants with limited tobacco exposure, we excluded six influential observations. Retention of these data points in the logistic regression model substantially altered the association between urine Cd and periodontal disease (OR = 1.05; 95% CI, 0.67–1.64). We provide a detailed discussion of our rationale for the identification of outliers and their characteristics [see Supplemental Material for details (http://www.ehponline.org/members/2009/0800312/suppl.pdf)].

## Discussion

In the present study, we found that environmental Cd exposure, as measured by creatinine-corrected urine Cd concentration, was associated with increased odds of prevalent periodontal disease in U.S. adults. In examining this association, we adjusted for smoking and several other established risk factors for periodontal disease that may potentially confound its observed relation to Cd exposure. To the best of our knowledge, this is the first epidemiologic study to report on the potential association between environmental Cd exposure and periodontal disease in U.S. adults.

Our findings are supported by the known effects of Cd exposure on bone and other tissues. Chronic low-level Cd exposure has been linked to decreased BMD and osteoporosis in a number of epidemiologic studies (Åkesson et al. 2006; Alfvén et al. 2000; Honda et al. 2003; Staessen et al. 1999). These results have been confirmed in animal experiments where administration of low levels of Cd over periods of up to 24 months resulted in decreased BMD and increased incidence of osteoporosis (Brzóska and Moniuszko-Jakoniuk 2004). In these experiments, the serum concentration of cross-linking telopeptide of type 1 collagen (a marker of bone resorption) was increased whereas the activity of serum alkaline phosphatase (a marker of bone formation) was decreased, indicating a disturbance of normal bone metabolism due to Cd exposure. Data from a number of laboratory experiments suggest that inflammatory mediators, such as prostanoids, cytokines, and MMP, may be responsible for these effects. *In vitro* experiments have shown that Cd exposure stimulates the production of prostaglandin E2 in osteoblast-like cells (Suzuki et al. 1989). Similarly, Cd promotes the release of calcium from organ cultures of neonatal mouse calvaria, and this effect is dependent on the induction of the prostaglandin-synthesizing enzyme cyclooxygenase-2 (Romare and Lundholm 1999). The effects of Cd on cytokines appear to be dose dependent such that low concentrations of Cd increase the release of cytokines (e.g., interleukin-1 and TNF-α), but at higher concentrations this effect is less prominent (Marth et al. 2000).

The available evidence thus suggests that Cd may further aggravate disturbances in the bone remodeling process that are present in periodontal disease. Periodontal disease is caused by accumulation of plaque bacteria in the gingival sulcus and subsequent release of lipopolysaccharide and microbial peptides that elicit a host inflammatory response (Preshaw et al. 2004). With advancing disease, there is destruction of periodontal ligament and loss of attachment caused primarily by host-derived enzymes (MMP) and inflammatory mediators (prostaglandins and cytokines). The latter are also responsible for the stimulation of osteoclasts, which leads to resorption of alveolar bone. Given the important role of inflammation in periodontal disease, it is plausible that environmental factors, such as exposure to Cd, that stimulate the release of inflammatory mediators may contribute to periodontal-disease–related tissue destruction.

Tobacco smoke contains many chemicals that may elevate the risk for periodontal disease (Barbour et al. 1997), and it is also a major source of Cd exposure in U.S. adults (Paschal et al. 2000). We therefore placed particular emphasis on distinguishing the relationship between Cd and periodontitis from the effects of smoking. We adjusted our analyses for cigarette smoking using self-reported smoking behavior and serum cotinine, a biomarker of exposure to tobacco smoke. We also used questionnaire-recorded responses to identify participants with exposure to cigarette smoke in their home and work environment. The relationship between creatinine-corrected urinary Cd and periodontal disease remained significant after we introduced these variables into our multivariable-adjusted logistic regression model. Without the inclusion of all tobacco variables in our analyses, the association between urinary Cd and periodontal disease was notably stronger than that reported in Table 2, confirming the importance of tobacco exposure in our study. Furthermore, we were concerned that tobacco exposure may contain several risk factors for periodontal disease that may partially “compete” with Cd, obscuring the observed association between Cd and periodontal disease among tobacco users and those exposed to high levels of ETS. It is possible that, among these individuals, other constituents of tobacco smoke may promote the development of periodontal disease to such an extent that Cd exposure no longer has a substantial impact on risk. We therefore undertook separate analyses among participants with very low tobacco exposure and found that the relationship between urinary Cd concentrations and periodontal disease was stronger in this group than among our full study population. However, this association was strongly influenced by six observations that markedly affected the parameter estimates for the Cd variable in our regression analyses. We therefore recommend caution in interpreting the association between Cd and periodontal disease observed in low-tobacco–exposed participants because this was statistically significant only upon removal of three influential points (and we also excluded three additional outliers). Although these observations represent a small proportion of cases in the low-tobacco–exposed subgroup [see Supplemental Material for details (http://www.ehponline.org/members/2009/0800312/suppl.pdf)], it is plausible that some residual confounding by tobacco exposure remained in our analyses and accounts for some or all of the association between Cd and periodontal disease.

Environmental lead exposure was also a potentially important confounder in our study because blood lead levels have been associated with periodontal-disease–related bone loss in this population (Dye et al. 2002; Saraiva et al. 2007). Furthermore, lead exposure has been linked with increased expression of MMP-9 (Barbosa et al. 2006), a protease that is also elevated in periodontal disease (Söder et al. in press). In addition to adjusting for blood lead concentrations in our main analyses, we undertook additional analyses restricted to participants with blood lead concentrations < 3 μg/dL and found that the association between urine Cd and periodontal disease was stronger.

The present study is limited primarily by its cross-sectional design, which prevents us from establishing a temporal relationship between Cd exposure and periodontal disease. However, the use of urinary Cd, which is a marker of long-term exposure, allowed us to consider the effects of Cd exposure over periods on the order of a decade or longer (Lauwerys et al. 1994). Furthermore, it is unlikely that reverse causality (periodontal disease affecting levels of Cd in urine) is responsible for the observed findings. In cross-sectional studies that include older adults, as this one does, survival to be in the cohort may be influenced by the exposure of interest; if Cd exposure increases the risk of mortality (Nawrot et al. 2008), any resulting bias in our findings would likely be toward underestimating Cd’s association with periodontal disease. Although our study is strengthened by a large sample size and the use of detailed measures of a number of important covariates, including exposure to tobacco smoke, unmeasured or mismeasured variables may have confounded the observed association between environmental Cd exposure and periodontal disease.

Widespread exposure and increasing evidence of systemic health effects at levels previously considered unimportant make environmental Cd exposure a significant public health issue. Although information on the oral health effects of this toxicant remains limited, our results suggest that Cd exposure may be an important risk factor for periodontal disease in adults. Prospective epidemiologic studies and controlled animal experiments are needed to confirm and elucidate the mechanisms behind the findings of this study.

## Figures and Tables

**Table 1:** Distribution of age-adjusted^a^ geometric mean urinary Cd concentrations by participant characteristics: NHANES III (1988–1994).

Characteristic	No. of participants^b^, ^c^ (unweighted)	μg Cd/g creatinine (95% CI)	Characteristic	No. of participants^b^, ^c^ (unweighted)	μg Cd/g creatinine (95% CI)
Periodontal disease^d^			Pack-years of cigarette smoking		
No	9,654	0.30 (0.28–0.31)	0	6,098	0.24 (0.23–0.26)
Yes	1,758	0.50 (0.45–0.56)	> 0–9.9	2,338	0.30 (0.27–0.32)
Sex			≥10	2,565	0.54 (0.51–0.57)
Male	5,602	0.26 (0.24–0.28)	No. of cigarette smokers at home		
Female	5,810	0.37 (0.35–0.40)	0	7,320	0.27 (0.25–0.28)
Age (years)^e^			≥1	4,082	0.42 (0.40–0.45)
18–29	3,460	0.15 (0.15–0.16)	Exposure to smoke at work		
30–39	2,671	0.29 (0.28–0.30)	No	4,435	0.27 (0.25–0.28)
40–49	1,990	0.39 (0.38–0.41)	Yes	2,885	0.33 (0.30–0.37)
50–59	1,042	0.49 (0.46–0.52)	Chewing tobacco		
≥60	2,168	0.57 (0.55–0.59)	Never-user	10,570	0.32 (0.30–0.33)
Education level of household head			Past user	385	0.32 (0.28–0.35)
< High school	2,251	0.35 (0.32–0.39)	Current user	273	0.28 (0.23–0.34)
High school	5,089	0.36 (0.33–0.39)	Pipe		
> High school	3,663	0.27 (0.26–0.29)	Never-smoker	10,656	0.31 (0.30–0.33)
Race/ethnicity			Past smoker	684	0.38 (0.34–0.42)
Non-Hispanic white	4,108	0.30 (0.28–0.32)	Current smoker	65	0.38 (0.24–0.59)
Non-Hispanic black	3,246	0.36 (0.35–0.38)	Cigars		
Mexican American	3,589	0.32 (0.31–0.34)	Never-smoker	10,348	0.32 (0.30–0.33)
Other	469	0.34 (0.29–0.39)	Past smoker	898	0.31 (0.28–0.35)
Household poverty status			Current smoker	161	0.28 (0.21–0.38)
< Federal poverty line	7,961	0.39 (0.36–0.43)	Blood lead [quartiles (μg/dL)]		
≥ Federal poverty line	2,454	0.30 (0.29–0.32)	< 1.7	2,969	0.27 (0.25–0.29)
Time since last visit to dentist (years)			1.7–2.8	2,853	0.28 (0.26–0.31)
≤ 1	7,424	0.30 (0.29–0.32)	2.9–4.6	2,737	0.33 (0.30–0.35)
> 1	3,927	0.33 (0.31–0.36)	> 4.6	2,836	0.41 (0.39–0.44)
Missing teeth			Diabetes		
None	3,856	0.25 (0.23–0.27)	No	9,940	0.31 (0.29–0.33)
1–10	6,630	0.35 (0.33–0.37)	Yes	1,357	0.38 (0.35–0.42)
> 10	926	0.52 (0.47–0.58)	Renal dysfunction		
Serum cotinine, ng/mL			No (urine albumin ≤30 μg/mL)	10,148	0.31 (0.29–0.32)
< 0.05 (detection limit)	1,285	0.24 (0.21–0.27)	Yes (urine albumin > 30 μg/mL)	1,263	0.39 (0.35–0.43)
0.05–10	6,811	0.27 (0.26–0.29)	Bone mineral density [quartiles (g/cm^2^)]		
> 10–100	895	0.28 (0.25–0.32)	< 0.87	2,482	0.36 (0.33–0.40)
> 100–250	1,175	0.46 (0.41–0.50)	0.87–0.97	2,503	0.34 (0.32–0.37)
≥ 250	1,246	0.58 (0.54–0.62)	0.98–1.08	2,501	0.28 (0.26–0.30)
			> 1.08	2,514	0.26 (0.24–0.28)

a Age-standardized to the 2000 U.S. census population using age categories of 18–39, 40–59, and > 60 years (Klein and Schoenborn 2001).

b Participants ≥18 years of age.

c Numbers of subjects differ because of missing data.

d Periodontal disease defined as having at least 4 mm of clinical attachment loss in > 10% of sites examined.

e Not adjusted for age.

**Table 2:** Multivariable-adjusted prevalence ORs for the presence of periodontal disease for a 3-fold increment^a^ in creatinine-corrected urine Cd concentration: NHANES III (1988–1994).

Logistic regression model	No. of participants^b^ (cases)	OR (95% CI)
Model type A: adjusted for systemic disorders and missing teeth^c^		
All participants		
Excluding five influential observations	5,585 (637)	1.54 (1.26–1.87)
Including all observations	5,590 (642)	1.32 (1.06–1.64)
Participants with limited tobacco exposure^d^		
Excluding six influential observations	1,575 (75)	1.68 (1.26–2.24)
Including all observations	1,581 (81)	1.05 (0.67–1.64)
Model type B: not adjusted for systemic disorders and missing teeth^e^		
All participants		
Excluding five influential observations	6,259 (676)	1.52 (1.25–1.85)
Including all observations	6,264 (681)	1.32 (1.09–1.62)
Participants with limited tobacco exposure^d^		
Excluding six influential observations	1,780 (79)	1.67 (1.26–2.21)
Including all observations	1,786 (85)	1.04 (0.72–1.50)

a A 3-fold increment in creatinine-corrected Cd concentrations is similar to the increment from the 25th percentile (0.18 μg/g) to the 75th percentile (0.63 μg/g) in urinary Cd concentration.

b Participants ≥ 18 years of age.

c Adjusted for sex, age, education level of household head, race/ethnicity, household poverty status, time since last visit to dentist, missing teeth, serum cotinine (log_e_ transformed), pack-years of cigarette smoking, number of cigarette smokers at home, exposure to smoke at work, chewing tobacco use, pipe smoking, cigar smoking, blood lead (log_e_ transformed), diabetes, renal dysfunction, BMD, examiner identification number, and survey phase.

d Participants, with serum cotinine concentrations ≤ 10 ng/mL, who reported never using cigarettes, chewing tobacco, cigars, or pipes and having no smokers at home and no exposure to cigarette smoke at work. We retained cotinine concentrations (log_e_ transformed) in the model.

e Adjusted for same variables as in model type A, except diabetes, BMD, and renal dysfunction, which may not be confounders but rather intermediates in the relation of Cd exposure to periodontal disease. We also did not adjust for missing teeth in this analysis because tooth loss may be a consequence of periodontal disease rather than a confounder.
